# Automatic characterization of stroke patients’ posturography based on probability density analysis

**DOI:** 10.1186/s12938-023-01069-z

**Published:** 2023-02-04

**Authors:** Ying Wang, Zhen Hu, Kai Chen, Ying Yang

**Affiliations:** 1 School of Mechanical Engineering, Hangzhou Dianzi University, Hangzhou, 310018 Zhejiang China; 2 Department of Neurology, Ruijin Hospital Luwan Branch Affiliated to Shanghai Jiao Tong University, Shanghai, 200000 China

**Keywords:** Postural control, Center of pressure, Probability density, Balance ability, Stroke patients

## Abstract

**Objective:**

The probability density analysis was applied to automatically characterize the center of pressure (COP) data for evaluation of the stroke patients’ balance ability.

**Methods:**

The real-time COP coordinates of 38 stroke patients with eyes open and closed during quiet standing were obtained, respectively, from a precision force platform. The COP data were analyzed and characterized by the commonly used parameters: total sway length (SL), sway radius (SR), envelope sway area (EA), and the probability density analysis based parameters: projection area (PA), skewness (SK) and kurtosis (KT), and their statistical correlations were analyzed. The differences of both conventional parameters and probability density parameters under the conditions of eyes open (EO) and eyes closed (EC) were compared.

**Results:**

The PA from probability density analysis is strongly correlated with SL and SR. Both the traditional parameters and probability density parameters in the EC state are significantly different from those in the EO state. The obtained various statokinesigrams were calculated and categorized into typical sway types through probability density function for clinical evaluation of the balance ability of stroke patients.

**Conclusions:**

The probability density analysis of COP data can be used to characterize the posturography for evaluation of the balance ability of stroke patients.

## Introduction

Balance and posture control play important roles in maintaining physical independence and flexibility [1]. Balance disorders and falls-related injuries are very common in stroke patients and may cause serious problems [2]. Stroke is caused by sudden death of brain cells, mainly because some brain cells die suddenly due to hypoxia when cerebral blood flow is blocked (cerebral infarction) or cerebral artery breaks (cerebral hemorrhage) [3], which is the second leading cause of death and the third leading cause of disability in the world [4]. After stroke, the brain of stroke patients cannot accurately perceive the information transmitted by the proprioceptive system of the whole body, resulting in dysfunction of the feeling of limb position and the actual movement control of limbs [5, 6]. Limb position sensory impairment means that there may be a loss of proprioception making the body unable to perceive the environment and body position information, while dysfunction of actual motor control of the limb means a complete loss or limitation of muscle control, mobility or activity level, often involving the patient’s upper and lower limbs unilaterally or bilaterally. Stroke patients usually have defects in motor control, which will lead to impaired balance and activity, resulting in a higher risk of falls [7, 8]. Therefore, accurate post-stroke assessment is extremely important for stroke patients. According to the assessment, effective interventions can be applied to reduce the susceptibility to falls and the possibility of falls [2].

The human’s balance ability during quiet standing is usually quantified by using a force platform to record the COP trajectory and generate so called statokinesigrams [9]. During quiet standing, human balance is achieved by constantly reconfiguring ground reaction forces under the feet to counteract the sway of the body [10]. The point of application of the vertical ground reaction force vector is known as center of pressure (COP) [10, 11]. The COP data are two-dimensional time series, and composed of the instantaneous position in the antero-posterior (AP) and medio-lateral (ML) directions. In order to quantify the balance ability and analyze the mechanism involved in postural adjustment, it is necessary to characterize the original COP data into useful variables [9, 12]. Baratto et al*.* proposed that COP variables can be divided into two basic categories: global variables and structural variables [13]. Global variables are used to estimate the overall size of the COP trajectory, including the trajectory length, envelope area, trajectory range, etc. [14]. It is generally believed that the larger the size or deviation of global variables, the worse the postural stability [11]. However, global variables are insensitive to the structure of variation, which may potentially provide insights into the posture control process in various environments [13]. Several studies have introduced scaling exponents that numerically define the structure of the COP traces instead of the mean magnitude of its variations [15]. Furthermore, the scaling exponent of COP has high intertest reliability and is shown to be a good predictor for risk of falling in the older population [16]. The structural analysis identifies sub-unities in the posturographic data and correlates them with the motor control processes [17]. The structural analysis of the COP has been proposed by several authors, among them Collins and De Luca [18], Baratto et al. [13], and Duarte and Zatsiorsky [19]. The parameters proposed by Collins et al. are based on the computation of “diffusion plots” or “variograms” [18]. The underlying idea of this approach is to model the stabilograms as fractional Brownian motions and thus to decompose the sway patterns into two stochastic processes: a short-term process, interpreted as an open-loop control mechanism, and a long-term process, interpreted as a closed-loop mechanism. The structural analysis proposed by Baratto et al. is based on a concept named sway-density curve [13]. The fundamental idea is that the postural stabilization is accomplished by the feedforward mechanism and so, the process of control is based on a sequence of anticipatory motor commands. The sway-density curves are built by counting the number of consecutive samples of the COP trajectory that fall within a circle of known radius. The typical sway-density plot presents regular alternating peaks and valleys: the peaks correspond to time instants in which the ankle torque and the associated motor commands are relatively stable; the valleys correspond to time instants in which the ankle torque rapidly shifts from one stable value to another. Zatsiorsky and Duarte have identified the presence of what they call “rambling” and “trembling” components in the stabilogram [19]. Rambling reflects the control ability of the nervous system and is the main component of body sway, whereas trembling reflects spinal reflexes and changes in the intrinsic mechanical properties of the muscles and joints [20].

Recent studies have found that the global parameters of the stroke group (COP elliptical swing area and COP sway path length) are significantly larger than those of the healthy control group [21], indicating that the static balance of the stroke group is seriously reduced and shows obvious postural instability, which is consistent with previous research results [22]. Rahimzadeh et al. analyzed the COP of stroke patients standing with eyes open (EO) and eyes closed (EC), and found that the sway range and sway speed of COP increased significantly during visual deprivation, indicating that the balance dysfunction caused by central nerve system damage was worse in the EC state, and also suggesting that the visual system played an important role in the balance control of stroke patients [23]. Wang et al. also conducted relevant experiments on stroke patients, and similar conclusions are obtained [24]. Nardone et al. reported that the stroke group showed greater values of area and length of COP displacement than healthy controls [25]. Although various global parameters have been used for characterization of the statokinesigrams, their clinic applications are restricted by their different definitions and interpretations. According to the stochastic theory, the information of COP position is all contained in its probability density function.

This study analyzes the COP data through the theory of probability density function. It is used to describe the probability distribution of continuous random variables. The probability density is defined as the probability within an interval divided by its length [26]. The probability density of COP is the probability of the COP occurring in a particular area of COP histogram. The collected COP data in a given time interval are in two-dimensional space (in *x* and *y* directions). Density is the number of the COP data within the small rectangle dx × dy divided by the area, when dx and dy approaching to zero. Due to the digital approximation, we counted the sample data falling into a minimum rectangle and then divided by its area to obtain the probability density. We actually have a two-dimensional histogram instead of a density. We demonstrate that the variables obtained through the probability density analysis are highly correlated with the conventional global variables, and can be used to quantify the stroke patients’ balance ability [27]. More importantly, the recorded statokinesigrams can be categorized into different sway types through the probability density analysis, which is useful in clinical applications.

## Results

### COP characteristic parameters and probability density diagram of typical participants

**Fig. 1:**
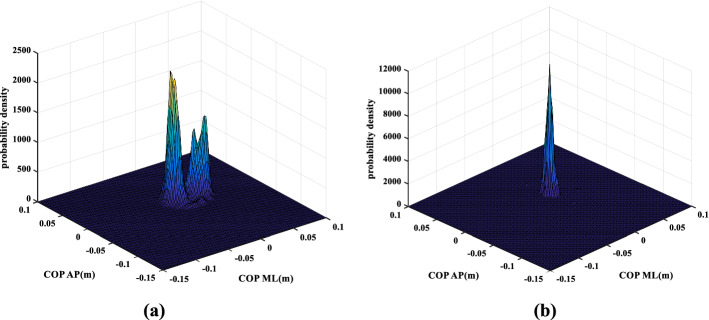
Two-dimensional COP probability density of typical participants. **a** Participant No. 5; **b** participant No. 30

The COP probability density diagrams of two participants with obvious different balance abilities are presented in Fig. 1. The stroke patients caused by occlusion or stenosis of internal carotid artery and vertebral artery generally need to undergo the Romberg test, which assesses the patient’s ability of balance and coordination [28]. Participant No. 5 had a positive sign of Romberg test due to internal carotid artery stenosis which often leads to weak balance ability. Participant No. 30 only felt a slight weakness in his right limb, and his balance ability was relatively good. Therefore, participant No. 5 and participant No. 30 were selected as typical participants for comparative analysis. The clinical characteristics and calculated COP characteristic parameters of participant No. 5 and participant No. 30 are listed in Table 1. It can be seen that there are significant differences of all the calculated COP parameters between participant No. 5 and participant No. 30. Participant No. 5 has significant larger values of total sway length (SL), sway radius (SR), envelope sway area (EA), projection area (PA, the size of the area enclosed by the outer contour projected by the two-dimensional probability density map of the COP trajectory to the AP-ML plane*)* and skewness (SK) in both ML direction (${\text{SK}}_{x}$) and AP direction (${\text{SK}}_{y}$), whereas significant smaller values of kurtosis (KT) in both ML direction (${\text{KT}}_{x}$) and AP direction (${\text{KT}}_{y}$), than those corresponding values of participant No. 30. The results imply that participant No. 5 has less balance ability than participant No. 30. Figure 1 shows the two-dimensional probability density diagram of COP data of participants No. 5 in Fig. 1a and No. 30 in Fig. 1b at EO state, respectively. The X and Y axes represent the coordinates of COP positions at a certain time in the ML and AP directions, respectively, and the *Z* axis represents the probability density value of COP at this position. It can be seen that participant No. 30 in Fig. 1b has only one high concentrated peak of probability density, whereas participant No. 5 in Fig. 1a has two relatively divergent peaks of probability density.

**Table1:** Comparison of basic characteristics and balance parameters of participants No. 5 and No. 30

	Participant No. 30	Participant No. 5
Characteristics		
Sex	Male	Male
Age	66	63
Internal carotid artery	Normal	Stenosis (bilateral severe)
Romberg sign	Difficult to cooperate	Positive
Limb muscle tone	Slight weakness of right limb	Normal
Time from stroke onset to test (days)	27	39
Balance parameters		
SL (m)	0.5220	1.3280
SR (m)	0.0048	0.0204
EA (cm^2^)	3.8113	29.0499
PA	24.9890	61.3090
SK_*x*_	0.1937	0.8799
SK_*y*_	0.0224	0.4691
KT_*x*_	6.5960	1.3876
KT_*y*_	5.4470	2.9511

*SL* total sway length, *SR* sway radius, *EA* envelope sway area, *PA* projection area, *SK* skewness, *KT* kurtosis

### Correlation analysis with conventional COP parameters

**Fig. 2:**
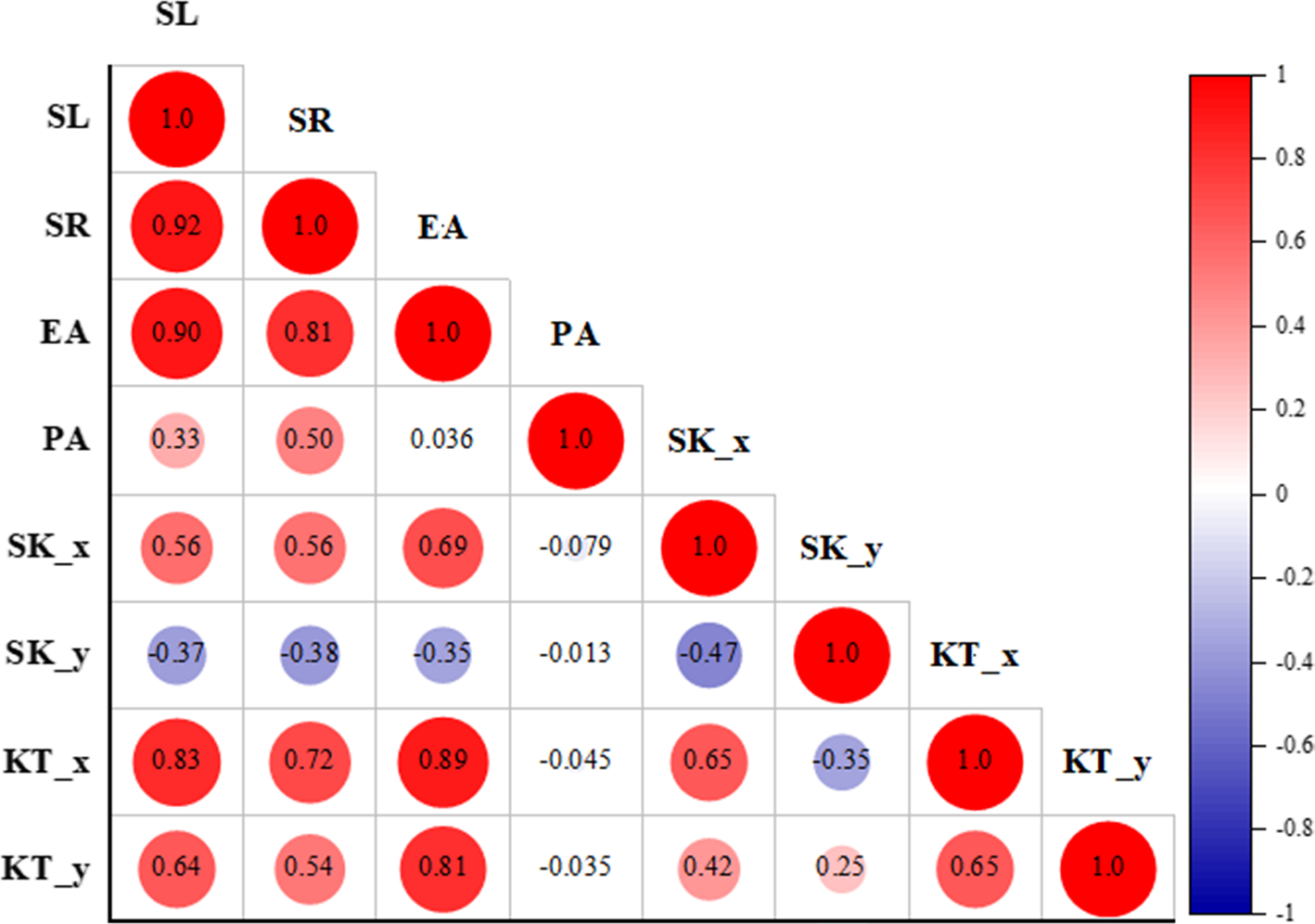
Correlation matrix between traditional parameters and probability density parameters. *SL* total sway length, *SR* sway radius, *EA* envelope sway area, *PA* projection area, *SK* skewness, *KT* kurtosis

The correlation analysis was performed for the entire group (38 participants). As shown in Fig. 2, under the EO condition, the total swing length (SL) of COP has a low correlation with the projected area (PA) of COP probability density (*r* = 0.33). There is a significant positive correlation between the sway radius (SR) of COP and the projected area (PA) of COP probability density (*r* = 0.50). However, there is no significant correlation between the trajectory envelope area (EA) of COP and the projected area (PA) of probability density (*r* = 0.036). ${\text{SK}}_{x}$ (in ML direction) is significantly positively correlated with the SL (*r* = 0.56), SR (*r* = 0.56) and EA (*r* = 0.69) of COP. ${\text{SK}}_{y}$ (in AP direction) is negatively correlated to SL (*r* = − 0.37), SR (*r* = − 0.38) and EA (*r* = − 0.35) of COP, but the correlations are not high. ${\text{KT}}_{x}$ (in the ML direction) is significantly and positively correlated with SL, SR and EA of COP (*r* = 0.83; *r* = 0.72; *r* = 0.89). ${\text{KT}}_{y}$(in AP direction) is positively correlated with SL, SR and EA of COP (*r* = 0.64; *r* = 0.54; *r* = 0.81).

### Comparison of COP parameters under different visual conditions

**Fig. 3:**
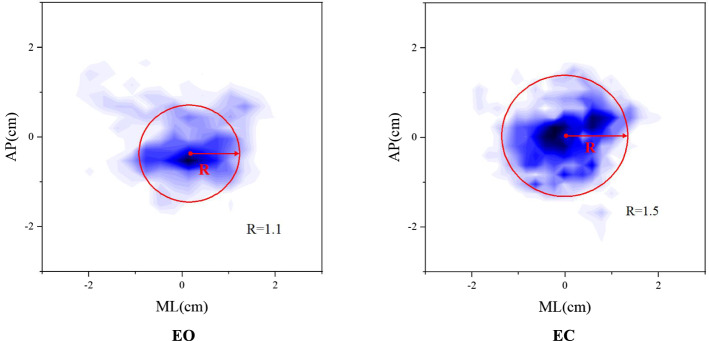
Comparison of probability density projections and radius of equivalent circle *R* of the participant No. 36 with different visual conditions: EO; EC

Paired t-tests were performed on the COP parameters of the 36 participants (except for participants No. 16 and No. 29) under different visual conditions, including the conventional variables: SL, SR and EA, and the variables based on probability density analysis: PA, ${\text{SK}}_{x} ,$ ${\text{SK}}_{y} ,$ ${\text{KT}}_{x} ,$ ${\text{KT}}_{y} .$ The calculation results are listed in Table 2. It shows that under different visual conditions, the differences between all COP variables except ${\text{SK}}_{y}$ are statistically significant. Figure 3 shows the probability density projection of COP of a participant in the EO state and EC state, respectively. The COP sway range is represented by the radius of equivalent circle (*R*), which is defined as the circle with the same area as the probability density projection area, and its center located at the maximum probability density. Obviously, the sway range of the participant’s COP trajectory in the EC state is larger than that in the EO state, which indicates the balance ability is reduced when vision is blocked.

**Table 2:** COP variables of participants in the state of EO and EC (paired *t*-test)

	Test conditions (including 36 participants)		*p*
	EO (mean ± SD)	EC (mean ± SD)	
SL (cm)	110 ± 10.1	130 ± 30.3	0.005**
SR (cm)	0.5 ± 5.6	0.7 ± 7.5	0.025*
EA (cm^2^)	15.8 ± 1.6	23.4 ± 1.9	0.014*
PA	53.6 ± 2.0	68.3 ± 2.4	0.002**
${\text{SK}}_{x}$	1.1 ± 0.3	1.5 ± 1.1	0.050*
${\text{SK}}_{y}$	0.9 ± 1.3	1.1 ± 1.5	0.09
${\text{KT}}_{x}$	11.3 ± 1.2	4.3 ± 0.6	0.000**
${\text{KT}}_{y}$	9.5 ± 0.6	4.2 ± 1.6	0.000**

*SL* total sway length, *SR* sway radius, *EA* envelope sway area, *PA* projection area, *SK* skewness, *KT* kurtosis, *SD* standard deviation

*p < 0.05, **p < 0.01

### Statokinesigram classification

In addition to COP variables, the statokinesigram generated by the COP trajectory is also useful for clinical diagnosis and evaluation of balance control. By visual observation of the statokinesigram, the features of COP sway direction, sway amplitude and its distribution in AP and ML directions can be judged subjectively. Statokinesigrams are generally classified into medio-lateral type, antero-posterior type, multi-centered type, diffusive type and sphere-centered type [29].

**Fig. 4:**
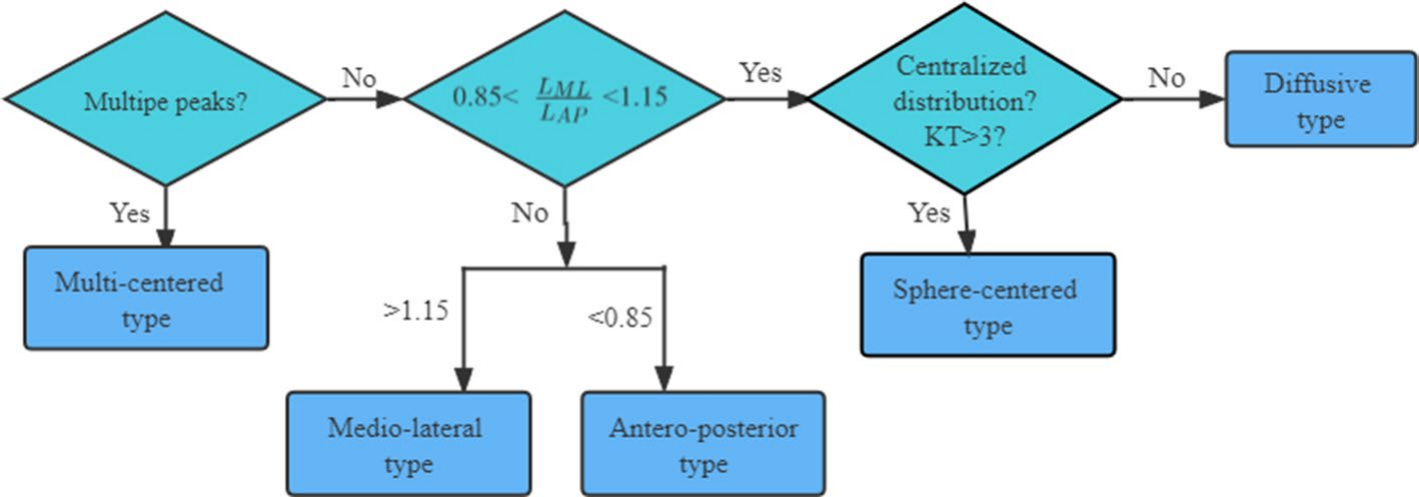
Flowchart of classification of sway types

**Fig. 5:**
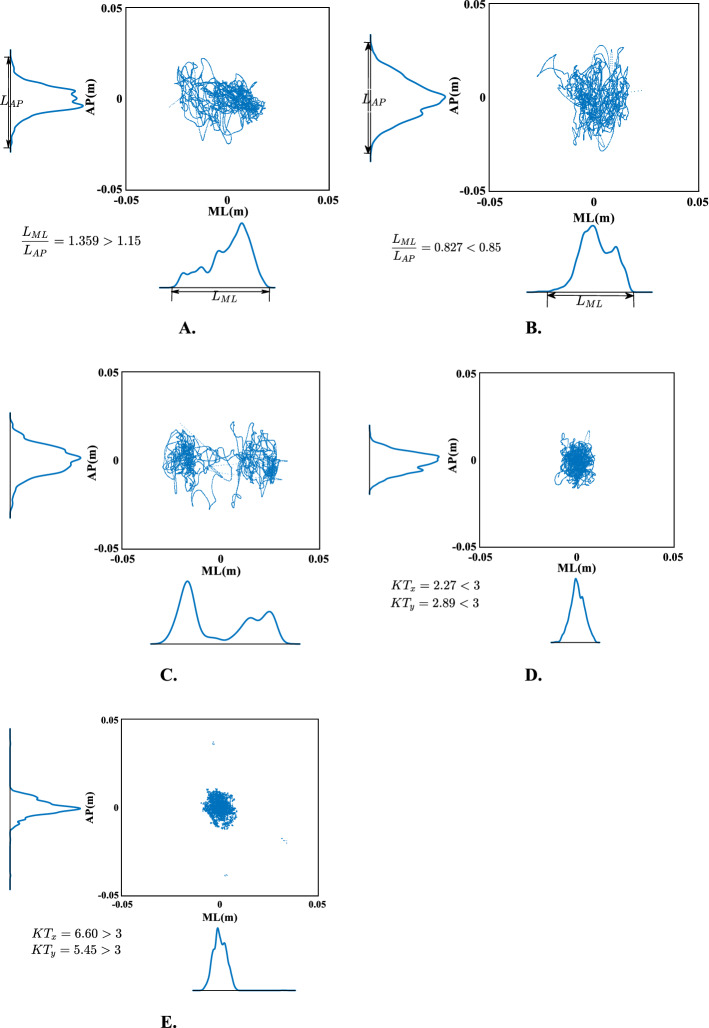
Five categorized types of statokinesigrams: **A** medio-lateral type, **B** antero-posterior type, **C** multi-centered type, **D** diffusive type, **E** sphere-centered type

Here, we present a quantitative approach for classification of statokinesigrams based on the COP probability density analysis. COP trajectory data were collected with the participant's eyes open. The logic steps of automatic classification are as follows: firstly, judge whether there are multiple peaks in the probability density diagram of COP. If there are multiple peaks, the sway type is multi-center type, otherwise proceed to the next step. In order to distinguish the medio-lateral type and the antero-posterior type, it is necessary to calculate the length of the 95% confidence interval. Taking the ML direction as an example, it means that the probability of the COP trajectory appearing in this interval in the ML direction is equal to 95%, and the length of this interval is called the length of the 95% confidence interval in the ML direction ${L_{\text{ML}}}$. Similarly, length of the 95% confidence interval in AP direction ${L_{\text{AP}}}$ can be obtained. If ${L_{\text{ML}}} /{L_{\text{AP}}} < 0.85,$ the participant’s statokinesigram type is of antero-posterior; if ${L_{\text{ML}}} /{L_{\text{AP}}} > 1.15,$ the statokinesigram type is of medio-lateral; otherwise, proceed to the next step. Next calculate the KT of the COP probability density. If ${\text{KT}} < 3,$ the statokinesigram type is of diffusive; otherwise, it is sphere-centered. The classification of statokinesigram based on the COP probability density analysis is thus completed. The cut-off values 0.85 and 1.15 were chosen based on our experience to be able to easily discriminate between the AP type and the ML type by observation. The flowchart is shown in Fig. 4. Figure 5 shows the categorized types of the typical participant’s statokinesigrams. Figure 5A, B, C, D, E are medio-lateral, antero-posterior, multi-centered, diffusive, and sphere-centered type, respectively. The main diagram is the statokinesigram of a particular participant, with ${L_{\text{AP}}}$ shown in the left and ${L_{\text{ML}}}$ indicated below. All the participants’ statokinesigrams were classified into five types through the aforementioned quantitative method, and the results are shown in Table 3.

**Table 3:** Classification of participants’ statokinesigrams

Statokinesigram	Medio-lateral type	Antero-posterior type	Multi-centered type	Diffusive type	Sphere-centered type
Patient number	2, 6, 12, 17, 21, 23, 38	3, 10, 11, 13, 15, 16, 19, 25, 26, 27, 31, 33, 34, 37	5, 8, 9	1, 4, 14, 18, 22, 24, 28, 29, 32, 35	7, 20, 30, 36

## Discussion

Postural instability is a common behavior of patients with neurological diseases such as stroke and Parkinson’s disease [30, 31]. Objective metrics for characterization of postural stability are necessary for the development of treatment strategies to aid rehabilitation [31]. The current research proposes a new method based on probability density of COP to evaluate the balance ability of stroke patients. The results show that variables retrieved from probability density analysis of COP signals can not only evaluate the postural stability of stroke patients, but also classify the statokinesigrams of stroke patients, so as to help doctors judge the balance ability of patients more intuitively and efficiently.

The results show that the projection area of the COP probability density in stroke patients is correlated with the total sway length and sway radius of COP trajectory under the static standing and EO state. Previous studies have shown that the balance ability can be objectively assessed through the total sway length of COP [32] and the sway range [33]. The projection area of COP probability density thus can be hypothetically used to characterize the balance ability of stroke patients. The envelope area of COP trajectory was also used to characterize the balance ability of patients with Parkinson’s disease [34, 35]. Kurtosis is the characteristic number that represents the peak height of the probability density distribution curve at the average value. In this study, kurtosis in both ML and AP directions are found strongly correlated with the envelope area of COP trajectory (*r* = 0.89; *r* = 0.81, respectively). Skewness is another statistical value that describes the distribution form of probability density function. The greater the absolute value of skewness, the greater the degree of dispersion on one side of the numerical mean [36]. Skewness in both ML and AP directions is found to be correlated with the total sway length and envelope area of COP trajectory in stroke patients.

It is worth pointing out that the COP position is a random variable. The traditional global variables, such as the length of trajectory, sway radius, sway area, etc., have been used for posturographic characterization. However, those variables can only reveal some information of the COP signals. For example, the sway radius is only related to the COP data point that is furthest from the center. The sway area has multiple definitions: both envelope area covering all data points, and the area covering certain confidence of data points have been used. Theoretically, the COP probability density function contains all the information of COP signals, and can describe the COP posturography more comprehensively. The high correlation between the probability density indexes and the traditional COP variables in the current study reflects the rationality and applicability of the probability density indexes.

In the experiments of different visual conditions, it is found that the COP sway range, total sway length, envelope area, as well as the projection area of probability density of COP of stroke patients are significantly increased if the vision is blocked; and the peak value of the probability density of COP is decreased, indicating that the extreme sway of COP is more in the closed eye state. All the above indicate that the balance regulation of stroke patients needs visual participation. The current results are also consistent with previous results [23, 24].

The statokinesigram can reflect the sway displacement size, distribution range and sway direction of patients on the force platform. Path length quantifies the magnitude of the two-dimensional displacement based on the total distance travelled. It is considered to be a valid outcome measurement in numerous populations and balance conditions—the smaller the path length, the better the postural stability [37]. The envelope area of COP trajectory is determined by a closed envelope curve that includes the majority of COP points. The smaller the envelope area, the better the postural stability [38]. The sway direction of COP signal can be intuitively obtained from the statokinesigram, as shown in Fig. 5. The variables retrieved from probability density analysis can be used to classify the statokinesigram into different types. Different statokinesigram types were found corresponding to balance disorders in different positions and organs of the central nervous system [29]. The sphere-centered type indicates that there is no balance disorder, which is no different from that of healthy in the meaning of the balance control [39]. The patients with unilateral labyrinthine disorder showed medio-lateral type due to the difference between left and right labyrinthine muscle tension. Spinal cord hyperreflexia caused by loss of labyrinth function in patients with bilateral labyrinthine disorder shows antero-posterior sway. Due to the damage of the vermis and middle part of the cerebellum, the coordinated movement disorder of the trunk and limbs is more characterized by diffusive sway. Multi-centered sway type often indicates that there are system damages in visual, auditory and proprioceptive systems, accompanied by cerebellar damage [29, 39]. In this study, participant 5 is a typical stroke patient, with hemorrhagic cerebral infarction (right cerebellum) and internal carotid artery stenosis (bilateral and severe), corresponding to multi-centered type. It should be pointed out here that similar statokinesigram results can be for many diseases. Only the comparison of the measurements for various disease entities can show the sensitivity and the specificity of this method to stroke. Therefore, in the following research, we will analyze the statokinesigram classification of other neurological diseases.

## Conclusion

Based on the COP trajectory data extracted from the force measuring platform, the current research proposed a method based on the COP probability density analysis to characterize postural balance of stroke patients. The variables retrieved through probability density analysis were compared with the conventional COP variables. The statistical correlation analyses show that the probability density variables have high correlations with the conventional COP variables. In addition, a method was proposed based on the COP probability density analysis to classify statokinesigrams of stroke patients into five types. The method provides a theoretical basis for the automatic and quantitative classification of COP signals.

## Materials and methods

### Participants

The participants of this balance ability test were 38 stroke patients in the Department of Neurology of Luwan branch of Ruijin Hospital, including 28 men and 10 women, with an average age of 63 years, whose body had poor balance ability due to different degrees of nervous system injury. Participants in the stroke group were discharged from in-patient rehabilitation within a month prior to testing. The study excluded subjects with cardiovascular diseases, tumor, neurological disorders except stroke, musculoskeletal injuries of lower limbs, severe malnutrition or severe visual defect. The participants’ clinical and demographic features are presented in Table 4. Two of the participants (except for No. 16 and No. 29) were excluded in doing COP measurement experiment under the stage of eyes-closed, considering their medical conditions. The test was approved by the ethics committee of Luwan branch of Ruijin Hospital (Approval No. lwec2019017) and agreed by the patients or their family.

**Table 4:** Demographic data of the participants

Characteristic, mean ± SD	Strokes (*N* = 38)
Age (years)	63.6 ± 4.9
Gender (female %)	73%
Weight (kg)	64.4 ± 3.2
Height (cm)	170.5 ± 5.5
Side of hemiparesis, left/right	29/9
Hemorrhage/infarction	5/33
Disease duration (days)	38.2 ± 5.8
BBS	47.0 ± 3.2

*BBS* Berg Balance Scale

### Data collection

In this study, the real-time coordinates of the participants’ COP were measured with the force measuring platform of AMTI (BP400600, Advanced Mechanical Technology Inc., MA, USA). The sampling frequency was 500 Hz, and the data were filtered by a zero-delay fourth-order Butterworth filter with a cut-off frequency of 8 Hz.

**Fig. 6:**
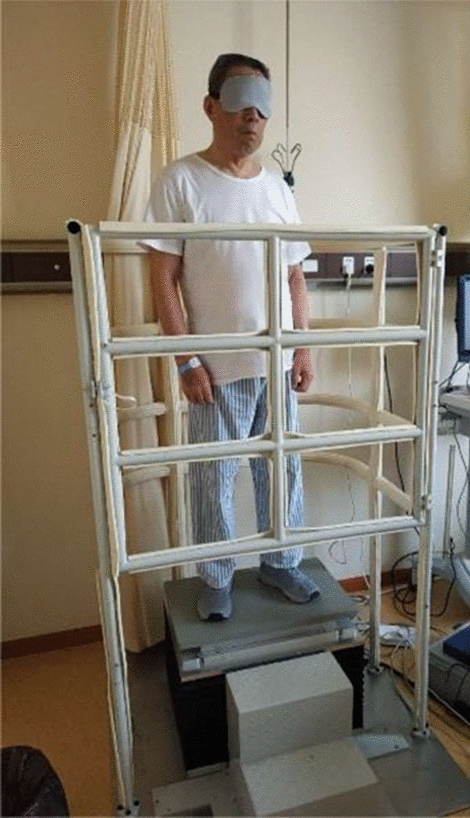
The participant tested on the force measuring platform

The test was conducted in a quiet environment. The participants were required to stand upright on the horizontal force measuring platform in the best posture, with their hands naturally hanging on both sides of their thighs, and their feet shoulder-width apart. During the EO test, the subjects were asked to gaze at the spot on the front wall, which was about 2.5 m apart and about the same level with the eyes. During the EC test, they wear eye masks to isolate visual feedback, as shown in Fig. 6. It should be noted that there are safety guardrails around the force platform to protect the participants from falling from the force platform. And during the experiment, there was an assistant beside the test platform to help the patient to step on the force platform and keep paying attention to the patient. If the patient had difficulty maintaining balance, the experiment was terminated. The balance test was repeated three times for each participant, and the average value was taken during the parameter calculation. In each test, the participant first performed the eyes-open test, and then rested for one to two minutes before performing the eyes-closed test. The test period for both eyes-open test and eyes-closed test is 50 s. In order to prevent the platform interference before and after the test, the collected data of the first 5 s and the last 5 s were deleted. The interval between each test was kept at least 3 min.

### Data processing and statistical analysis

MATLAB R2016a was used for experimental data analysis. In the data processing and calculation, the *x*-axis was the medio-lateral (ML) direction of subjects, which was parallel to the frontal plane; and the *y*-axis was the antero-posterior (AP) direction, which was perpendicular to the frontal plane. Due to the different standing positions of patients in each test, the starting points of COP on the force measuring platform were different in each measurement. In order to ensure the consistency of subsequent data analysis, we took off the offset of the COP data by subtracting the average position in the two directions as the sway starting point, i.e., the origin of the coordinate system. We assumed that the recorded COP trajectory contained ${n}$ data points, for each $1 \le i \le n,$

1 $${x_{i}} = {x^{\prime}} -{\overline{x}},$$

2 $${y_{i}} = {y^{\prime}} - {\overline{y}},$$

where ${x^{\prime}}$ and ${y^{\prime}}$ represent the coordinates of COP position at a certain time in the ML and AP directions, respectively. $\overline{x}$ and $\overline{y}$ are the average value of coordinates of all data points. ${x_{i}}$ and ${y_{i}}$ represent the corrected coordinates of COP position at a certain time in the ML and AP directions, respectively.

IBM SPSS statistics 26.0 was used for statistical analysis, and Pearson correlation analysis was used for correlation analysis. The significance levels of all statistical analyses were set as *p < 0.05, **p < 0.01.

#### Calculation of traditional parameters

The traditional global COP parameters including total sway length (SL) [9], sway radius (SR) and trajectory envelope area (EA) were calculated. The calculation formulas of SL and SR were as follows:

3 $${\text{SL}} = \sum\limits_{i = 1}^{n} {\sqrt {\left( {{x_{i + 1}} - {x_{i}} } \right)^{2} + \left( {{y_{i + 1}} - {y_{i}} } \right)^{2} } } ,$$

4 $${\text{SR}} = \frac{1}{n}\sum\limits_{i = 1}^{n} {\sqrt {\left( {{x_{i}} - {\overline{x}}} \right)^{2} + \left( {{y_{i}} - {\overline{y}}} \right)^{2} } } .$$

**Fig. 7:**
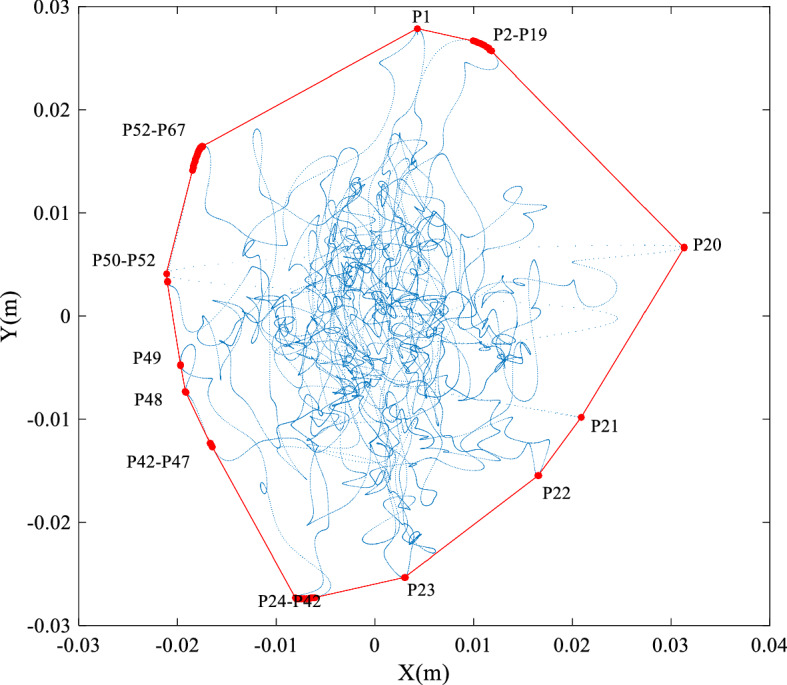
Schematic diagram of convex hull of COP trajectory

Trajectory envelope area (EA) referred to the envelope area of the COP coordinate data collected within the specified time. Researcher has proposed a method to obtain EA as follows [38]: first search the outer contour points of COP trajectory, then connect these outer contour points in turn to form a convex polygon containing all COP data, as shown in Fig. 7, where ${P_{1}} ,{P_{2}} \ldots$ are the convex hull of the coordinate point set. After obtaining the coordinates of the convex hull vertex, the envelope area can be calculated through the combined triangulation method:

5 $${S_{i - 2}} = \frac{1}{2}\left| {\left( {{x_{1}} {y_{i - 1}} - {y_{1}} {x_{i - 1}} } \right) + \left( {{x_{i - 1}} {y_{i}} - {y_{i - 1}} {x_{i}} } \right) + \left( {{x_{i}} {y_{1}} - {y_{i}} {x_{1}} } \right)} \right|,$$

where EA is calculated from $i = 3$ until the convex point set is searched. Finally, the value of ${S_{i - 2}}$ is accumulated to obtain the total envelope area.

#### Calculation of parameters by probability density function

Several parameters retrieved from probability density analysis were applied in the current research: the projected area (PA) of COP probability density diagram, the skewness (SK) and kurtosis (KT) of COP marginal probability density in *x*-axis and *y*-axis directions. Firstly, the COP probability density was obtained by kernel density estimation, which was a method used to estimate unknown density function in probability theory. The kernel density of ${x_{1}} ,{x_{2}} \cdots {x_{n}}$ which were independent identically distributed sample points was estimated as [40]:

6 $${\hat{f}_{h}} (x) = \frac{1}{n}\sum\limits_{i = 1}^{n} {{K_{h}} } (x - {x_{i}} ) = \frac{1}{nh}\sum\limits_{i = 1}^{n} {K\left( {\frac{{x - {x_{i}} }}{h}} \right)} .$$

For two-dimensional data, kernel density was estimated as:

7 $${\hat{f}_{h}} (x,y) = \frac{1}{{nh^{2} }}\sum\limits_{i = 1}^{n} {K\left( {\frac{{{\text{dist}}\left( {\left( {x,y} \right),\left( {{x_{i}} ,{y_{i} }} \right)} \right)}}{h}} \right)} ,$$

where $K( \cdot )$ is a kernel function, and Gaussian kernel function is used here; $h$ is bandwidth; ${K_{h}} (x) = \frac{1}{h}K\left( \frac{x}{h} \right)$ is scaled kernel function, where ${\text{dist}}\left( {\left( {x,y} \right),\left( {{x_{i} },{y_{i}} } \right)} \right)$ is the Euclidean distance between $\left( {x,y} \right)$ and $\left( {{x_{i}} ,{y_{i}} } \right).$

Projection area is the area of two-dimensional projection of COP probability density plot in ML-AP direction. After the COP probability density function was obtained, PA can be calculated by binarization method in Matlab. And the ‘graythresh’ function can help us obtain a suitable threshold using the maximum inter class variance method.

SK and KT in ML(*x*)-direction can be calculated by the following formula [41]:

8 $${\text{SK}}_{x} = \frac{1}{n}\sum\limits_{i = 1}^{n} {\left[ {\left( {\frac{{{x_{i}} - \mu }}{\sigma }} \right)^{3} } \right]} ,$$

9 $${\text{KT}}_{x} = \frac{1}{n}\sum\limits_{i = 1}^{n} {\left[ {\left( {\frac{{{x_{i}} - \mu }}{\sigma }} \right)^{4} } \right]} ,$$

where ${n}$ is the number of samples, ${\mu}$ is the sample average in ML(*x*)-direction, and $\sigma$ is the standard deviation in ML(*x*)-direction. Replace the $x_{i}$ in Eqs. (8) and (9) with $y_{i}$ to get the corresponding ${\text{SK}}_{y}$ and ${\text{KT}}_{y}$. SK is the measure of the skew direction and degree of samples distribution. The smaller the absolute value of SK, the more symmetrical the samples distribution is. KT is the characteristic number of the peak height of the probability density distribution curve at the average value. The larger KT indicates that the sample distribution is more concentrated and the less extreme samples at both ends. In particular, SK = 0, KT = 3 indicates that the samples are in normal distribution [42].

## Data Availability

All relevant data are within the paper, and please visit the following URL for its Additional information: 10.6084/m9.figshare.20367303.v2.
